# Key Challenges to the Effective Management of Pollutants in Water and Sediment

**DOI:** 10.3390/toxics10050219

**Published:** 2022-04-27

**Authors:** Fátima Jesus, Louis A. Tremblay

**Affiliations:** 1 CESAM and Department of Environment and Planning, University of Aveiro, Santiago Campus, 3810-193 Aveiro, Portugal; 2 Cawthron Institute, Private Bag 2, Nelson 7042, New Zealand; 3 School of Biological Sciences, University of Auckland, Auckland 1142, New Zealand

The intensification of human activities is placing increasing pressure on the ecosystems of riverine, estuarine, and coastal waters, as these compartments are sinks for many anthropogenic contaminants [1]. A multiscale spatial model analysis demonstrated that no marine ecosystem remains unaffected by the adverse influence of human activities [2], and freshwater ecosystems are also under pressure (e.g., [3]). Using a boundaries framework to assess the integrity of Earth system processes, it was recently concluded that humanity is currently operating at levels beyond our planet’s capacity to sustain life [4].

There has been much effort to assess the effects of aquatic pollution, given the importance of healthy water to sustain life. A search of the Scopus database with the keywords “aquatic AND toxicity” retrieved about 25,000 papers. The number of papers increased from 671 in 2010 to 2449 in 2021. Despite the increasing research on contaminants’ impact on exposed biota, many knowledge gaps and challenges in fully assessing the risk of contaminants remain.

The number and diversity of chemicals produced have never been greater. Traditionally, research on aquatic pollution has focused on metals, organic compounds such as polycyclic aromatic hydrocarbons, wastewater effluents, and pesticides [5]. In recent decades, there has been increasing interest in contaminants of emerging concern, including nanomaterials, pharmaceuticals and personal care products, and microplastics [6,7,8]. With more than 100 million chemical compounds registered in the Chemical Abstracts Service (CAS), and roughly 4000 new chemicals registered daily [9], assessing their risk is paramount. Studying and predicting the potential effects of these chemicals on exposed biota highlight the key role of ecotoxicology in protecting the receiving ecosystems. In addition to the increasing number of chemicals released into the environment, there is a need to address the risk of complex mixtures [10]. Indeed, synergistic interactions between chemicals can occur [11]. Not all combinations of chemicals can be studied, and future research should focus on the most probable contaminant mixtures to improve the risk assessment and better inform and support environmental managers and regulators.

Research on the ecotoxicity of chemicals in sediment has been overlooked. For instance, considering the abovementioned bibliographic search, only 8.5% of papers considered sediments. Benthic species are key receptors, as sediments are often contaminant sinks, particularly the most hydrophobic ones (e.g., [12,13]). Sediment toxicity is driven by the bioavailability of contaminants and partitioning in porewater [13]. Bioavailable contaminants may accumulate in benthic biota, reaching higher concentrations than sediment [12]. Bioaccumulation raises concerns about the potential trophic transfer to higher organisms which might, ultimately, pose a risk to human health.

This Special Issue, “Ecotoxicity of Contaminants in Water and Sediment”, attempts to address some of the key challenges facing contaminant risk assessors and managers. Here are some of the challenges and knowledge gaps that we have identified to better characterize the risk that contaminants pose to receiving ecosystems:

1. The need to consider the expanding number of chemicals and impacts of multiple stressors: In addition to the increasing number of chemicals entering the environment, one of the key challenges remains risk assessment of complex mixtures [14,15]. The risk can be further compounded by other stressors, including those related to climate change and global population growth [16].
2. Better frameworks for the risk assessment of contaminants in sediments are required: A wide variety of chemical stressors can accumulate in sediment, and it is important to address their potential effects to aquatic biota, particularly benthic species [17,18].
3. The risk assessment frameworks and approaches need to consider the diversity of the receiving aquatic ecosystems: The standardization of conditions for ecotoxicological assessment has limitations in its ability to appropriately address the wide range of diversity and the conditions of the sites being investigated. For instance, there should be some flexibility, considering the wide distribution of soft waters [19] and the differences in the sensitivity of native species compared to standard test species (e.g., [20]).
4. The importance of extending ecotoxicological assessment beyond acute exposure: Further assessments of the toxicity of multiple stressors should incorporate sublethal effects following chronic exposure, transgenerational effects, and trophic transfer to underpin acute toxicity results [21,22]. These aspects contribute to an integrative comprehension of contaminants’ toxicity, thus supporting informed decisions on their environmental management for the protection of both the environment and human health.
5. Finally, there is a need to focus on solutions: There is no doubt that the accumulation of anthropogenic pollutants in the environment is causing harm and scientists need to work with other stakeholders to reduce pollution. We need to better manage the use of chemicals in society to find the best balance between their benefit and the level of potential adverse environmental effects [23]. The management and regulation of chemicals should be underpinned by the principle of “do no harm”, while ensuring the full benefits of their use [24]. The solutions should also consider societal and cultural values [25].
